# Pharmaceuticals wastage and pharmaceuticals waste management in public health facilities of Dessie town, North East Ethiopia

**DOI:** 10.1371/journal.pone.0259160

**Published:** 2021-10-28

**Authors:** Solomon Ahmed Mohammed, Mesfin Haile Kahissay, Abel Demerew Hailu

**Affiliations:** 1 Department of Pharmacy, College of Medicine and Health Science, Wollo University, Dessie, Ethiopia; 2 Department of Pharmacy, Dessie Health Science College, Dessie, Ethiopia; Nobel College Pvt Ltd, NEPAL

## Abstract

**Background:**

Pharmaceuticals wastes are drugs and medicines that can no longer be used. The improper disposal of unused medicines is a growing problem throughout the world. This study assessed the pharmaceutical wastage rate and pharmaceutical waste management for the year 2015 to 2017 in the public health facility of Dessie, Ethiopia.

**Methodology:**

A cross-section study design was used to review logistic data retrospectively from health commodity management information systems and manual records in 8 health facilities. Health professionals’ (135) pharmaceutical waste management practices were assessed using the world health organization waste management checklist. Descriptive and inferential statistics were made using a statistical package for social sciences version 20.

**Result:**

Supplies were the leading class of pharmaceuticals with an overall wastage rate of 37.1%. Tablet and injectable constituted the highest class of pharmaceuticals dosage form with the overall wastage rate of 20.78% and 16.49%. The overall pharmaceutical wastage rate was 3.68% amounting to USD 159,762.66 and expiry (92.05%) was the major reason for wastage. The pharmaceutical wastage rate of health centers was nearly twofold higher than hospitals. Pharmaceutical waste management was practiced by 105 (77%; 95% CI; 69.9%, 84.9%) health professionals. Determinants of pharmaceutical waste management were being male (P value = 0.08, AOR = 3.72), receiving training (P value = 0.01, AOR = 4.34), writing label (P value = 0.02, AOR = 5.04), storage of segregated waste in dispensing unit (P value = 0.01, AOR = 0.72) and the presence of disposal plan (P value = 0.002, AOR = 16.93).

**Conclusions:**

Supplies and tablets constituted the highest wastage class of pharmaceuticals and dosage form. The pharmaceutical wastage rate was higher than the standard and increasing in successive years. Pharmaceutical waste management was not fully practiced. Appropriate inventory control and waste management are recommended.

## Background

Pharmaceuticals wastes are drugs that can no longer be used because of being expired, unused, spilled, withdrawn, recalled, damaged, contaminated, or for any other reason [1, 2]. It also includes discarded items heavily contaminated during the handling of pharmaceuticals, such as bottles, vials, and boxes containing pharmaceutical residues, gloves, masks, and connecting tubing [3].

Pharmaceuticals are used in health care facilities to save millions of lives by preventing and treating diseases and improve the quality of life of the human population [4]. Medicines account for 20–60% of health spending in developing and transitional countries [5]. Incorrect inventory management, lengthy procurement cycles, poor storage, improper monitoring of drug expiration times, distribution problems, and irrational usage of drugs results in wastage of pharmaceuticals [6]. This resulted in wastage of financial resources, shortage of essential medicines, increase in out-of-pocket expenditure, and decline in quality of healthcare services [7].

In the United States of America, approximately 2 of 3 prescription medications were reported unused and the total estimated cost was approximately 59,264.20 USD (United States dollar) to 152,014.89 USD [8]. In Palestinian hospitals, the percentages of unused drug products that expired, or those with no clear expiry date were 32.7%, 17.7%, and 11% respectively. The most common drug categories encountered in households were alimentary, musculoskeletal, and anti-infective agents whereas the most common individual drugs encountered were: paracetamol (8.5%), ibuprofen (4.9%), and diclofenac (3.7%) [9]. A study of medicines wastage at a tertiary hospital in Dar Es Salaam showed that 730 medicines dosages were wasted. Anti-infective medicines wastage was 18.9%, cardiovascular medicines (8.9%) and the other categories were 23.7% of the total medicines dispensed [10]. Other studies in rural Ugandan hospitals revealed that expired drugs were worth 1584 USD (25 items) in 2000/2001 and 1307 USD (13 items) in 2004/2005 [11]. In Ethiopia, the average wastage rate was reported to be 1.1% in auditable pharmaceutical and transaction systems implementing hospitals and it accounted for 3,196,865 birrs by value in 2014/2015 [12]. Another study on Gonder town showed that the total loss of money due to expiry over six months was 1337.6 USD from six health facilities [13].

Waste management includes waste collection, packaging, storage, segregation, transport, treatment, and disposal [14] and needs to be done on a more scientific basis [15]. Hospital waste management is a significant environmental and social obligation, and hence requires a proper plan [16]. Pharmaceutical waste should be segregated from other wastes [17] and the amount of generated waste should be forecasted for the planning of waste management budgets and optimization of waste management practices [18].

Globally, all health care services generate 3% of pharmaceuticals waste [19, 20]. Today their disposal is alarming as they generate large quantities of waste and by-products [21]. The improper disposal of unused medicines is a growing problem throughout the world [22]. In developing countries, proper disposal of hazardous wastes is still a significant challenge [23] and there were no established medication waste management programmers [24]. A study in Jordan showed that the handling, storage, and disposal of generated wastes have less appropriate practices as compared to the developed world, and management of hazardous or general wastes is below acceptable medical waste standards [25].

Pharmaceutical wastes are potentially generated through a wide variety of activities in the health care system [21]. However, their increased consumption has entailed a health risk due to the increased loading of pharmaceutical discharge and waste into the environment during consumption and disposal [26]. Poor management of health care waste can cause serious problems to health care personnel, waste workers, patients, and the general public [19]. Higher risk of medical wastes associated with lower knowledge about waste separation compromised the health care delivery [27]. A study conducted in Jordan showed that there is a statistically significant impact between environment and efficiency and effectiveness of medical waste separation, waste classification, waste collection, and storage in hospitals [28].

Taking the aforementioned issues into consideration, this research was attempted to provide an answer to the following basic questions. What was the pharmaceuticals wastage rate of public health facilities of Dessie town for the year 2015 to 2017? How was the pharmaceuticals waste management practice of public health facilities of Dessie town? In the attempt of answering the research’s questions, the following hypothesis was constructed and was tested for its significance: Is there an increment in the annual pharmaceuticals wastage rate. Is there a statistically significant relationship between pharmaceuticals waste management and socio-demographic characteristics of professionals.

There was no similar study conducted in Dessie town. Despite few studies were done elsewhere in the world, they can’t be applied to the local context due to differences in study settings. This study would help health institutions and decision makers’ effort on proper ways of pharmaceuticals waste reduction and management to optimize scarce resources and safeguarding the health of healthcare providers, clients, and the general population. Therefore, this study assessed pharmaceuticals wastage rate and pharmaceuticals waste management in public health facilities of Dessie town.

## Methods

### Study area and period

**Fig 1:**
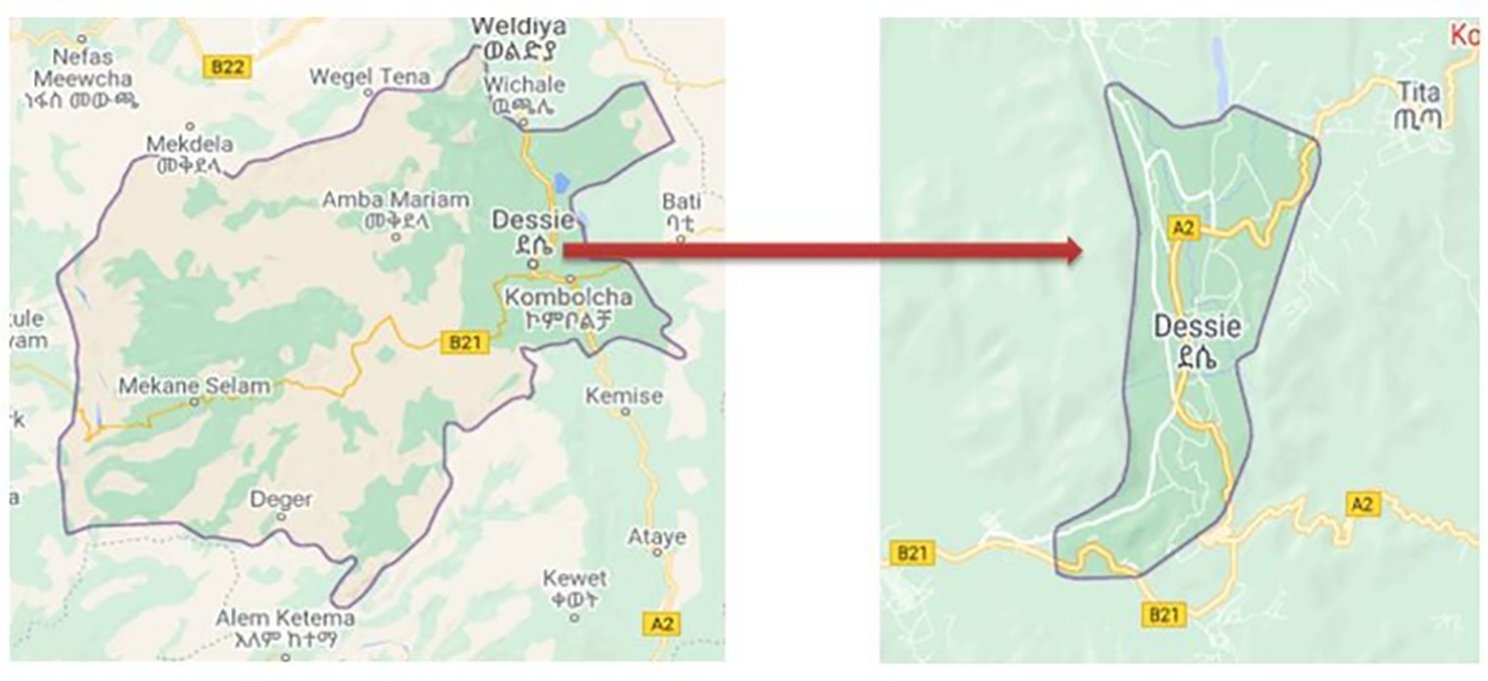
Map of Dessie town (source; Google map, Available at: https://www.google.com/search?q=south+wollo+map&source=lmns&bih=654&biw=1366&client=firefox-b e&hl=en&sa=X&ved=2ahUKEwjyyfmwgMryAhUBahoKHbDRCq0Q_AUoAHoECAEQAA#).

A study was conducted from November 2018—February 2019 in the public health facility of Dessie town, Ethiopia. Dessie (Fig 1) is located in the South Wollo zone of Amhara National Regional State, 401 kilometers away from Addis Ababa, the capital city of Ethiopia. According to the 2017 national census, Dessie has a total population of 151,174 [29]. In Dessie town, there are 8 public health centers, one referral hospital, and a district hospital serving Dessie town and the surrounding nearly 8 million populations.

### Study design

Cross-sectional study design which required retrospective data collection was used to review logistic data from bin cards, stock cards, and health commodity management information systems. It was also used to administer questionnaires for all professionals working in pharmacy stores and dispensing units.

### Study population

All received and wasted revolving drug fund pharmaceuticals in public health facility of Dessie town between 2015–2017 and all professionals working in pharmacy store and dispensing units. A total of 10 public health facilities (2 hospitals and 8 health centers) were surveyed but 2 health centers were excluded due to incomplete data on the extent of pharmaceutical wastage. Wasted pharmaceuticals recorded as free price such as program and donation medicines and outside the stated years were excluded from the study.

A total of 145 self-administered questionnaires were distributed to health professionals who engaged in pharmaceutical store management and dispensing units and the overall response rate was 93%.

### Data collection instruments

A structured checklist adopted from Logistics Indicator Assessment Tool (LIAT) [30] and world health organization (WHO) waste management checklist [31] was used to collect data on pharmaceutical wastage rate and contributing factors, and waste management respectively. Pharmaceutical wastage data were abstracted by the principal investigators by reviewing receiving the voucher, health commodity management information system, bin cards, and stock cards. Two pharmacists who had no working relation to the health institutions used a self-administered questionnaire to collect the data under the supervision of the principal investigators.

### Data management and analysis

Data were entered and analyzed using Microsoft Excel 2010 and Statistical Package for Social Sciences version 20. Forward binary logistic regressions were used and crude and adjusted odds ratio was calculated with 95% confidence intervals and variables with p-value <0.05 were taken as statistically significant. Pharmaceuticals were classified according to their pharmacotherapeutic classes using the classification of medicines adopted from the Ethiopian national essential medicine list [32]. The structured data collection tools were properly designed for the assurance of data quality. All data were examined for completeness and consistency during data collection, analysis, and interpretation.

### Ethical considerations

Ethical approval was obtained from the Ethics Review Committee of the college of medicine and health sciences, Wollo University (phar 037/11), and Dessie town health bureau. Then, the study was conducted after getting permission from each respective public health facility. Study participants were informed about the purpose of the study and verbal informed consent was obtained from study participants. In this study, verbal informed consent was approved by the ethics review committee and confidentiality of study participants’ data was maintained throughout the study.

## Results

### The magnitude of pharmaceutical wastage

Public health facilities of Dessie town received United States Dollar (USD) 1,860,116.76 in 2015 and the annual wastage rate was 2.7% amounting to USD 50,269.90 (Table 1).

**Table 1:** Pharmaceutical wastage rate in public health facilities of Dessie town, Ethiopia, (n = 8).

Sr. no	Code of health facilities	2015			2016			2017		
		Received USD	Wastage USD	% wastage	Received USD	Wastage USD	% wastage	Received USD	Wastage USD	% wastage
**1**	DHC	33,830.15	4,490.09	13.27	32,678.79	2,450.80	7.50	29,200.43	487.08	1.67
**2**	SGHC	15,183.69	435.42	2.87	16,610.66	562.92	3.39	16,656.51	3,128.18	18.78
**3**	BHC	5,888.73	136.48	2.32	8,128.56	288.73	3.55	8,701.83	734.10	8.44
**4**	KHC	5,925.17	1,220.38	20.60	8,430.60	587.90	6.97	9,501.35	268.29	2.82
**5**	GHC	9,718.33	167.76	1.73	9,043.96	585.45	6.47	9,915.43	1,215.39	12.26
**6**	MHC	3,507.54	241.88	6.90	3,507.54	452.45	12.90	3,507.54	751.29	21.42
**7**	BMH *	100,754.72	1,159.49	1.15	210,562.53	8,383.74	3.98	272,074.50	17,845.76	6.56
**8**	DRH *	1,685,308.43	42,418.40	2.52	845,764.62	35,349.86	4.18	994,303.66	36,400.83	3.66
**Average**		1,860,116.76	50,269.90	2.70	1,134,727.27	48,661.83	4.29	1,343,861.25	60,830.92	4.53

* Hospital.

The three-year pharmaceutical wastage analysis revealed that the overall wastage rate was 3.68% (2.70%, 4.29%, and 4.53% in 2015, 2016, and 2017 respectively) amounting to USD 159,762.66. Excluding hospitals from analysis, 7.92% of the wastage rate was found in six health centers amounting to USD 18,204.59. The major reasons for wastage of pharmaceuticals were expiry (92.05%), damage (9.94%), and lost (0.02%) amounting USD 128,151.04, 11,047.28 and 23.11 respectively.

A total of 449 pharmaceuticals were assessed and supplies were the leading class of pharmaceuticals with the overall wastage rate of 37.1% accounting for USD 51,937.43 while the list was recorded in vaccine with a wastage rate of 0.01% amounting to USD 20.40 (Table 2).

**Table 2:** Pharmaceuticals wastage rate by class in public health facilities of Dessie town, Ethiopia, (n = 8).

Sr. no	Classes of pharmaceuticals	Wasted value USD	% wastage
**1**	Anti-infectives	32,415.73	23.28
**2**	Anesthesia medicine	5,865.04	4.21
**3**	Anti-histamines	911.59	0.65
**4**	Blood products/ Anti-anemic	4,056.68	2.91
**5**	Central nervous system	10,242.78	7.36
**6**	Cardiovascular	3,375.18	2.42
**7**	Dermatological agents	930.25	0.67
**8**	Endocrine	4,145.50	2.98
**9**	Ear-Nose- Throat preparations	4,534.47	3.26
**10**	Gastrointestinal	3,434.44	2.47
**11**	Obstetric/ Gynecological	1,728.23	1.24
**12**	Musculoskeletal	176.70	0.13
**13**	Respiratory	2,135.32	1.53
**14**	Vaccine	20.40	0.01
**15**	Vitamins	2,490.69	1.79
**16**	Water for injection	544.18	0.39
**17**	Water, electrolyte, and acid-base balance correcting	2,526.36	1.81
**18**	Laboratory reagents and chemical	7,750.45	5.57
**19**	Supply	51,937.43	37.31
**Total**		139,221.44	100.00

Tablet and injectable constituted the highest class of pharmaceuticals dosage form with the overall wastage rate of 20.78% and 16.49% amounting to USD 29,056.58, and 22,960.64 respectively (Table 3). Examination glove medium size, clarithromycin 500 mg tablet, and chloramphenicol 250 capsule took the highest wastage value amounting to USD 10,496. 02, 7627.54, and 6712.39 with the wastage rate of 7.54%, 5.48%, and 4.82 respectively from the total received pharmaceuticals.

**Table 3:** Pharmaceuticals wastage rate by dosage form in public health facilities of Dessie town, Ethiopia, (n = 8).

Sr. no	Dosage form	Wasted value USD	% wastage
**1**	Aerosols/ inhalation	584.29	0.42
**2**	Capsule	9,958.38	7.15
**3**	Cream	848.07	0.61
**4**	Drop	5,774.84	4.15
**5**	Elixir	223.02	0.16
**6**	Jell	101.38	0.07
**7**	Injection	22,960.64	16.49
**8**	Tablet	29,056.58	20.87
**9**	Lotion	108.38	0.08
**10**	Ointment	993.26	0.71
**11**	Pessary	154.32	0.11
**12**	Powder	5,367.42	3.86
**13**	Solution	277.88	0.20
**14**	Syrup	1,162.46	0.83
**15**	Suppository	864.78	0.62
**16**	Suspension	1,097.85	0.79
**17**	Supply	51,937.43	37.31
**18**	Laboratory reagents and chemical	7,750.45	5.57
**Total**		139,221.44	100.00

### Pharmaceutical waste management

There were 53.3% male respondents. The respondents consisted of 20 up to 56 years of age with a mean age of 29.36 (±6.37). With regards to the level of education, 44.4% were diploma while the rest 55.6% had the degree. Their work experience ranged from beginner to 31 years with a mean work experience of 6.28 (±5.44) (Table 4).

**Table 4:** Socio-demographic characteristics of health professionals in public health facilities of Dessie town, Ethiopia (n = 135).

Sr. no	Socio-demographic profile		Number	Percentage
**1**	Sex	Male	72	53.3
		Female	63	46.7
**2**	Age	20–29	83	61.5
		30–39	41	30.4
		≥40	11	8.1
**3**	Work experience	<5	52	38.5
		5–10	57	42.2
		>10	26	19.3
**4**	Level of education	Diploma	60	44.4
		Degree	75	55.6
**5**	Profession	Pharmacy	42	31.1
		Druggist	43	31.9
		Midwifery	11	8.2
		Health officer	5	3.7
		Nurse	26	19.2
		Laboratory technology	8	5.9
**6**	Training on pharmaceutical waste management	Yes	40	29.6
		No	95	70.4

Pharmaceutical waste management was practiced by 105 (77%; 95% CI; 69.9%, 84.9%) health professionals. Segregated pharmaceuticals stored in 35.6% dispensing units waiting for removal and 68.1% used cartons as a sort of container for segregation. Moreover, 66.7% of respondents revealed that pharmaceuticals wastes were disposed of within health facilities compounds and the usual (63.7%) methods of disposal were burning in the open air (Table 5).

**Table 5:** Pharmaceutical waste management practices in public health facilities of Dessie town, Ethiopia, (n = 135).

Sr. no	Descriptions		Number	Percentage
**1**	Container or bag used for segregation	Carton	92	68.1
		Plastic bag	19	14.1
		Wastebasket	24	17.8
**2**	Writing label on segregated waste	Yes	81	60
		No	54	40
**3**	Recording of segregated waste	Yes	90	66.7
		No	45	33.3
**4**	Storage of segregated waste awaiting removal	Dispensing unit	48	35.6
		Pharmacy store	63	46.7
		Both	24	17.8
**5**	Waste disposal guideline	Yes	87	64.4
		No	48	35.6
**6**	Waste disposal plan	Yes	79	58.5
		No	56	41.5
**7**	Waste disposal team	Yes	89	65.9
		No	46	34.1
**8**	Place of disposal	Municipal land	45	33.3
		Health facility compound	90	66.7
**9**	Frequency of disposal	Annually	103	76.3
		Quarterly	17	12.6
		Every six month	15	11.1
**10**	Method of disposal	Incineration	27	20
		Burning	86	63.7
		Landfill	22	16.3
**11**	Impact of waste disposal on the environment	Yes	92	68.1
		No	43	31.9

Forward binary logistic regression showed that male health professionals (P value = 0.08, AOR = 3.72) and health professionals who received training on pharmaceutical waste management (P value = 0.01, AOR = 4.34) have a statistically significant association on pharmaceutical waste management (Table 6).

**Table 6:** Socio-demographic factors associated with pharmaceutical waste management in public health facilities of Dessie town.

Sr. no	Socio-demographic profile	Pharmaceutical waste management		COR (95% CI)	AOR (95% CI)
		Yes	No		
**1**	Sex				
	Male	61(84.7)	11(15.3)	0.38(0.16–0.89)	3.72(1.4–9.85)
	Female	43(68.3)	20(31.7)	1	1
**2**	Age				
	20–29	67(80.7)	16(19.3)	1.57(0.37–6.59)	2.27(0.24–21.06)
	30–39	29(70.7)	12(29.3)	0.90(0.20–4.01)	1.03(0.14–7.3)
	≥40	8(72.7)	3(27.3)	1	1
**3**	Work experience				
	<5	41(78.8)	11(21.2)	1.97(0.69–5.62)	1.54(0.32–7.45)
	10-May	46(80.7)	11(19.3)	2.21(0.78–6.27)	2.34(0.56–9.77)
	>10	17(65.4)	9(34.6)	1	1
**4**	Level of education				
	Diploma	48(80)	12(20)	0.73(0.32–1.67)	3.44(0.54–21.59)
	Degree	56(74.7)	19(25.3)	1	1
**5**	Profession				
	Pharmacy	34(81)	8(19)	1.49(0.55–4.04)	1.77(0.52–5.99)
	Druggist	33(76.7)	10(23.3)	1.15(0.44–2.99)	0.26(0.37–1.83)
	Other*	37(74)	13(26)	1	1
**6**	Training on pharmaceutical waste management				
	Yes	36(90)	4(10)	0.28(0.09–0.86)	4.34(1.28–14.69)
	No	68(71.6)	27(28.4)	1	1

Other* Midwifery, health officer, nurse, laboratory technology.

The adjusted model indicated that health professionals who wrote a label on segregated waste (P value = 0.02, AOR = 5.04), storage of segregated of waste in dispensing unit (P value = 0.01, AOR = 0.72), and the presences of disposal plan (P value = 0.002, AOR = 16.93) has a statistically significant effect on pharmaceutical waste management (Table 7).

**Table 7:** Factors associated with pharmaceutical waste management in public health facilities of Dessie town, Ethiopia.

Sr. no	Descriptions	Pharmaceutical waste management		COR (95% CI)	AOR (95% CI)
		Yes	No		
**1**	Container or bag used for segregation				
	Carton	67(64.4)	25(80.6)	0.38(0.1–1.39)	0.3(0.04–2.08)
	Plastic bag	16(15.3)	3(9.7)	0.76(0.13–4.28)	0.31(0.02–3.5)
	Wastebasket	21(20.3)	3(9.7)	1	1
**2**	Writing label on segregated waste				
	Yes	73(90.1)	8(9.9)	0.14(0.06–0.36)	5.04(1.28–19.7)
	No	31(57.4)	23(42.6)	1	1
**3**	Recording of segregated waste				
	Yes	76(84.4)	14(15.6)	0.3(0.13–0.69)	1.36(0.4–4.63)
	No	28(62.2)	17(37.8)	1	1
**4**	Storage of segregated waste awaiting removal				
	Dispensing unit	34(70.8)	14(29.2)	0.81(0.26–2.46)	0.72(0.01–0.57)
	Pharmacy store	52(82.5)	11(17.5)	1.57(0.5–4.87)	0.2(0.02–1.51)
	Both	18(75)	6(25)	1	1
**5**	Waste disposal guideline				
	Yes	77(88.5)	1011.5)	0.16(0.17–0.39)	1.3(0.34–4.98)
	No	27(56.3)	2143.8)	1	1
**6**	Waste disposal plan				
	Yes	74(93.7)	5(6.3)	0.07(0.02–0.22)	16.93(2.71–105)
	No	30(53.6)	26(46.4)	1	1
**7**	Waste disposal team				
	Yes	80(89.9)	9(10.1)	0.12(0.05–0.3)	1.89(0.44–8.02)
	No	24(52.2)	22(47.8)	1	1
**8**	Place of disposal				
	Municipal land	29(64.4)	16(35.6)	2.75(1.2–6.29)	0.27(0.07–1.01)
	Health facility compound	75(83.3)	15(16.7)	1	1
**9**	Frequency of disposal				
	Annually	77(74.8)	26(25.2)	0.21(0.02–1.68)	0.68(0.04–10.3)
	Quarterly	13(76.5)	4(23.5)	0.23(0.02–2.35)	0.25(0.01–4.7)
	Every six month	14(93.3)	1(6.7)	1	1
**10**	Method of disposal				
	Incineration	15(55.6)	12(44.4)	0.19(0.04–0.82)	0.11(0.01–1.39)
	Burning	70(81.4)	16(18.6)	0.69(0.18–2.62)	0.89(0.14–5.47)
	Landfill	19(86.4)	3(13.6)	1	1
**11**	Impact of waste disposal on the environment				
	Yes	73(79.3)	19(20.7)	0.67(0.29–1.55)	0.47(0.11–2.01)
	No	31(72.1)	12(27.9)	1	1

## Discussion

Pharmaceuticals are used to prevent, treat and improve the quality of life of the human population in health facilities [4]. Because of their health value, about one-third of the budget is spent on purchasing various materials and supplies including medicines, and forty percent of the budget is spent on procurement & management of stores [33]. In Ethiopia, the total drug expenditure grew over years still now [12, 34].

The present study revealed that the overall wastage rate was 3.68% amounting to USD 159,762.66. The finding was 3.34 and 1.42 fold higher than the average wastage rate (1.1%) and value (USD 108,002) in auditable pharmaceutical and transaction systems implementing hospitals [12]. But the current study finding was lower than the South West Shoa Zone health facilities wastage rate (7.5%) [35].

A wastage rate of 7.92% was found in six health centers of Dessie town amounting to USD 18,204.59. The result was higher than the lost money from a similar number of health facilities in Gonder town (USD 1337.6) [13]. The present study result was slightly lower than the average rate of wastage (8.5%) in nine health centers amounting to USD 11,906.23 in health facilities of South West Shoa Zone [35]. The difference might be attributed to the number and type of health facilities and length of reviewed time included in the study.

On the other hand, wastage in two hospitals of Dessie town was USD 141,558.07 with an overall 3.45% wastage rate. This was 1.73 times lower than the wastage rate in one hospital of South West Shoa Zone (6%) with a value of USD 5,003.2 [35] but higher than rural Ugandan hospitals where expired drugs worth USD 1584 in 2000/2001 and USD 1307 in 2004/2005 [11]. The difference was due to the number of pharmaceuticals included in the study and the volume of hospitals.

This study revealed that the pharmaceutical wastage rate of health centers was nearly twofold higher than hospitals. This was in line with the study conducted in Serbia where tertiary healthcare level hospitals produced statistically significantly larger quantities of healthcare waste than secondary level hospitals [36]. The differences in wastage rate across various studies might be due to differences in duration of the study, assessed types, number and characteristics of health facilities, number of items, and inflation of the currency.

The overall wastage rate in the successive years was increasing and it was higher than the national standard (2%) wastage rate [37]. The amount of waste generated was increased as the number of patient flow increased [38]. The mean pharmaceutical waste generation was 0.017kg/day in the health centers of West Gojjam Zone [39] and 1.17 kg/day in Mizan Tepi University teaching hospital [40]. This results from a loss of scarce financial resources and extra resources needed for handling, processing, and disposal of pharmaceutical waste which impose a financial burden for health facilities as well as the country [7]. Further wastage resulted in a shortage of essential medicines, an increase in out-of-pocket expenditure, and a decline in the quality of healthcare services.

In the present study, the reasons for pharmaceuticals wastage were expiry (92.05%), damage (9.94%), and loss (0.02%). The result was nearly in line with the value of expired (95.7%), while three times higher in damaged value (3.1%) and no observed record of obsoleted (1.2%) [35]. Even though the magnitude of loss is minimal, pilferage was attempted. Wastage of 26.5% because of pilferage at a tertiary Hospital in Dar Es Salaam were factors contributing to medicines wastage [10]. This requires effective and efficient inventory management for the prevention of pilferage and damage [41, 42].

Anti-infectives (23.28%), central nervous system (7.36%), and anesthesia (4.21%) took a greater share of wastage value in the present study. Anti-infective medicines and cardiovascular medicines wastage was 18.9% and 8.9% in tertiary Hospitals in Dar Es Salaam [10] and Sweileh et al. study in Palestinian hospital showed that alimentary, musculoskeletal, and anti-infective agents were the most common categories [9]. The University hospital pediatric units of Brazil showed that 22.7% antimicrobials, 14.8% electrolytes, 14.6% analgesics/pain killers, 9.5% diuretics, and 6.7% antiulcer agents were wasted [43]. This might be attributed to health facilities in developing countries like Ethiopia were more prone to infectious disease as they demand more anti-infective. On the other hand, the reason for the inflated wastage rate of injectable might be due to perceived superior efficacy which letter increases purchase volume resulted from deviation in prescribing practices from the standard recommended by WHO [44]. Delivery of near expiry date drugs was also the main reason [45].

Nearly two-thirds of respondents did not take training on pharmaceutical waste management and having training increases pharmaceutical waste management by 4.34. The proportion of respondents who had received specific training was 11.5% in Nigeria [46], 40% in Khartoum state hospital [47], and low in Bangladesh [48]. Continuous training should be conducted and for further improvement, a larger number of trained specialists in the field is mandatory [36].

Proper management of pharmaceutical wastes requires separating and storing wastes at the point of generation. In the present study, nearly two and one-third of the respondents label (AOR = 8.37) and store (AOR = 0.72) on pharmaceutical wastes respectively, and this was significantly associated with pharmaceutical wastes management. The finding was better than hospitals in Addis Ababa [38], health centers in West Gojjam Zone [39], and low-level health facilities in Tanzania [49] where waste separation and treatment practices were poor. So, all healthcare facilities are obligated to separate, label, and safely put away and limit access to wastes from unauthorized personnel [36]. The storage area should be enclosed, labeled, and separated from other waste storage area [17].

The commonly used container or bag for segregation was a carton in the health facilities of Dessie town. Similarly, normal waste bins used for over 75% disposed of pharmaceuticals in selected hospitals of Ghana [50]. In the University hospital of Brazil, sharps’ disposable box with a yellow bag, sink drain, sharps box with orange bag, and infectious waste/bin with a white bag was used [43]. When planning storage of waste, the characteristics of the specific chemicals to be stored and disposed of must be considered [17] and all waste-bag seals should be in place and intact until they are transported to disposal sites [51].

This study found that the presence of a pharmaceutical waste disposal plan (AOR = 16.93) was statistically associated with waste management. There were no specific regulations or guidelines used for waste management in health centers in West Gojjam Zone [39], Ghana [50, 52], and most of the developing countries including Asia [24]. Similarly, 55% of hospitals in Khartoum state have no clear policy of waste management and only 20% have a waste management plan [53] while 9% and 47% of the healthcare facilities in Ilala and Kinondoni, respectively do not have the standard operating procedures [49]. As a result, waste management practice in Mizan Tepi University teaching hospital [40] and different hospitals of Jordan [25] was not coherent with the WHO guideline.

Burning was a widely practiced method of disposal. Hospitals of Addis Ababa dispose of through incineration [38], while health centers in West Gojjam Zone used local types of incinerators and open burn [39]. Most of the pharmaceuticals in the present study were disposed of within the health facilities compound. This was in line with the disposal practice of hospitals of Addis Ababa [38] while most of the low-level facilities have no specific disposal sites in Tanzania [49]. Nowadays, pharmaceutical waste disposal generates large quantities of waste and by-products [21]. The explosion of toxic air pollutants due to open burning and dumping on uncontrolled sites may contaminate the environment or increase the chance of diversion of products to the market for resale and misuse [4] and proper disposal is still a significant challenge in developing countries [23].

Sixty-eight percent (68%) of respondents believed that the current pharmaceutical waste management and disposal had an impact on the environment. A study conducted in Jordan showed that there is a statistically significant impact between environment and efficiency and effectiveness of medical waste separation, waste classification, waste collection, and storage in hospitals [28]. Contamination of wastewater by antibiotics or other pollutants can lead to the rise of drug resistance [54]. As a result, pharmaceutical waste can harm patient’s or medical employee’s health, as well as the environment [36] and it is more beneficial to educate and improve practice regarding disposal of pharmaceutical waste [55].

A large number of pharmaceuticals are used every day and their use is on the rise. Effective and efficient pharmaceutical management in health institutions is a crucial factor for reducing waste and plays a greater role in the environmentally responsible disposal of pharmaceutical waste. Therefore, the study pinpointed to health institutions and decision-makers the extent of pharmaceutical wastage and waste management to optimize scarce resources and safeguarding the health of healthcare providers, clients, and the general population. The study utilizes recorded data from health facilities and the information management systems in health institutions may introduce bias to the study. Moreover, the cross-sectional nature of this study might make it harder to establish a temporal relationship.

## Conclusion

Supplies constituted the leading class of pharmaceuticals wastage. Tablet and injectable had the highest class of pharmaceuticals dosage forms wastage. The pharmaceutical wastage rate was higher than the standard wastage rate. The wastage rate was increasing in successive years and the expiry of pharmaceuticals was the major reason. The pharmaceutical wastage rate of health centers was nearly twofold higher than hospitals.

More than two-thirds of health professionals had practiced pharmaceutical waste management. Cartons were used as a sort of container for segregation and pharmaceuticals waste disposed of within health facilities compound and the usual method of disposal were burning in the open air. Determinants of pharmaceutical waste management were the sex of health professionals, receiving training on pharmaceutical waste management, writing a label on segregated waste, storage of segregated waste in dispensing unit, and the presence of disposal plan. Appropriate inventory management and the development and adoption of clear health facility-specific health care waste management plans and policies are recommended. The finding of this study will be used as input for researches. It will also help policymakers for developing interventional measures for reducing pharmaceutical wastage and execute proper pharmaceutical waste management practices.

## List of abbreviations

LIAT: Logistics Indicator Assessment Tool
USD: United States Dollar
WHO: World health organization

## References

[1] Nwachukwu NC, Orji FA, Ugbogu OC. Health care waste management–public health benefits, and the need for effective environmental regulatory surveillance in federal Republic of Nigeria. Current Topics in Public Health: InTech; 2013. doi: 10.5772/53196

[2] West LM. Medication wastage: the current situation. J Malta College of Pharmacy Practice. 2015;21:25–8.

[3] Chartier Y. Safe management of wastes from health-care activities: World Health Organization; 2014.

[4] International Committee of the Red Cross. Medical Waste Management. Geneva, Switzerland. November 2011. avaialble at: www.icrc.org/en/doc/assets/files/publications/icrc-002-4032.pdf

[5] Cameron A, Ewen M, Ross-Degnan D, Ball D, Laing R. Medicine prices, availability, and affordability in 36 developing and middle-income countries: a secondary analysis. v. The lancet. 2009;373(9659):240–9. 10.1016/S0140-6736(08)61762-6 19042012

[6] Romero A, editor Managing medicines in the hospital pharmacy: logistics inefficiencies. Proceedings of the World Congress on Engineering and Computer Science; 2013.

[7] Susan W, Joseph O. An Assessment of the Effects of Inventory Management Procedures on Performance of Kengen. International Journal of Scientific and Research Publications 2015; 5(10). Available at: www.ijsrp.org/research-paper-1015/ijsrp-p46122.pdf

[8] Law AV, Sakharkar P, Zargarzadeh A, Tai BWB, Hess K, Hata M, et al. Taking stock of medication wastage: unused medications in US households. Research in Social and Administrative Pharmacy. 2015;11(4):571–8. doi: 10.1016/j.sapharm.2014.10.003 25487420

[9] Sweileh WM, Sawalha AF, Zyoud S, Al-Jabi SW, Bani Shamseh F, Khalaf HS. Storage, utilization and cost of drug products in Palestinian households. International journal of clinical pharmacology and therapeutics. 2010;48(1):59. doi: 10.5414/cpp48059 20040340

[10] Kagashe GA, Makenya FB, Buma D. Medicines Wastage at a Tertiary Hospital in Dar Es Salaam Tanzania. Journal of Applied Pharmaceutical Science. 2014;4(6):98–102. doi: 10.7324/JAPS.2014.40615

[11] Tumwine Y, Kutyabami P, Odoi RA, Kalyango JN. Availability and expiry of essential medicines and supplies during the ‘pull’and ‘push’drug acquisition systems in a rural Ugandan hospital. Tropical Journal of Pharmaceutical Research. 2010;9(6).

[12] Teferi Gedif Fenta Bethelhem Gulelat, Assefa Tamrat, Teshome D. Outcomes of Auditable Pharmaceutical Transactions and Services (APTS) Implementation: Assessment Report. Submitted to the Federal Ministry of Health (FMOH), Ethiopian Pharmaceutical Association (EPA), and Systems for Improved Access to Pharmaceuticals and Services (SIAPS). Addis Ababa: FMOH, EPA, and SIAPS. 2016.

[13] Fentie M, Addisie Fenta FM, Oumer H, Belay S, Sebhat Y, Atinafu T, et al. Availability of Essential Medicines and Inventory Management Practice in Primary Public Health Facilities of Gondar Town, North West Ethiopia. Journal of Pharma Sci Tech. 2015;4:54–6.

[14] Rushton L. Health hazards and waste management. British medical bulletin. 2003;68(1):183–97. doi: 10.1093/bmb/ldg034 14757717

[15] Shalini S, Harsh M, Mathur B. Evaluation of bio-medical waste management practices in a government medical college and hospital. Natl J Community Med. 2012;3:80–4.

[16] Amin R, Gul R, Mehrab A. Hospital Waste Management; Practices in different hospitals of Distt. Peshawar. Professional Medical Journal. 2013;20(6).

[17] World Health Organization. Safe management of wastes from health-care activities: a summary. 2017.

[18] Gusca J, Kalnins SN, Blumberga D, Bozhko L, Khabdullina Z, Khabdullin A. Assessment method of health care waste generation in Latvia and Kazakhstan. Energy Procedia. 2015;72:175–9. doi: 10.1016/j.egypro.2015.06.025

[19] World Health Organization. Management of solid health-care waste at primary health-care centres: A decision-making guide. Management of solid health-care waste at primary health-care centres: a decision-making guide: WHO; 2005.

[20] World Health Organization. Waste from health-care activities. Fact Sheet no. 253. Geneva: World Health Organization. 2011 Nov.

[21] Kadam A, Patil S, Patil S, Tumkur A. Pharmaceutical Waste Management An Overview. Indian Journal of Pharmacy Practice. 2016;9(1):3. doi: 10.5530/ijopp.9.1.2

[22] Manojlović J, Jovanović V, Georgiev AM, Tesink JG, Arsić T, Marinković V. Pharmaceutical Waste Management in Pharmacies at the Primary Level of Health Care in Serbia-Situation Analysis. Indian Journal of Pharmaceutical Education and Research. 2015;49(2):106–11. doi: 10.5530/ijper.49.2.5

[23] Harhay MO, Halpern SD, Harhay JS, Olliaro PL. Health care waste management: a neglected and growing public health problem worldwide. Tropical Medicine & International Health. 2009;14(11):1414–7. doi: 10.1111/j.1365-3156.2009.02386.x 19735368

[24] Bataduwaarachchi VR, Weeraratne CL. Global medication waste management practices: challenges and opportunities in developing countries. International Journal of Basic & Clinical Pharmacology. 2018;5(6):2290–4. 10.18203/2319-2003.ijbcp20164081.

[25] Aditya S, Singh H. Safe medication disposal: Need to sensitize undergraduate students. International Journal of Pharmacy & Life Sciences. 2013;4(3).

[26] Becker J. Minding the gap: Research priorities to address pharmaceuticals in the environment. Health Care Without Harm, Health Care Research Collaborative Retrieved from https://saludsindanio.org/sites/default/files/documents-files/74/Minding_the_Gap_Research.pdf.

[27] Ferreira V, Ribau Teixeira M. Assessing the medical waste management practices and associated risk perceptions in Algarve hospitals. Portugal Google Scholar. 2009.

[28] Habash M, Zubi A. Efficiency and effectiveness of medical waste management performance, healthcare sector and its impact on environment in Jordan applied study. World Applied Sciences Journal. 2012;19(6):880–93. doi: 10.5829/idosi.wasj.2012.19.06.775

[29] Population Censeus Commission. Summary and statistical report of the 2007 population and housing census. Population size by age and sex. 2008.

[30] USAID Deliver Project. Logistics Indicators Assessment Tool (LIAT). Task Order. 2008. Arlington, VA: USAID.

[31] World Health Organization Regional Office for South-East Asia. Survey questionnaire for hospital waste management. Available at: www.bvsde.paho.org/bvsacd/infviaje/residuos/041923/041923…/questionaire.pdf doi: 10.1017/mdh.2017.34 28604290 PMC5471977

[32] EFMHACA. National Essential Medicine List. Fifth Edition, Addis Ababa. November, 2014. Available at: apps.who.int/medicinedocs/documents/s22351en/s22351en.pdf.

[33] **Renukadevi R. A study of inventory control practices in the department of stores of a tertiary care hospital, 2010. http://www.rguhs.ac.in/cdc/onlinecdc/uploads/15_HM07_25373.doc doi: 10.1039/c0mt00062k 21132183

[34] World Health Organization. Drug financing in Ethiopia. September 2007. Available at: http://www.who.int/medicines/areas/coordination/ethiopia_financing.pdf.

[35] Tadesse E. Assessment of medicines wastage and its contributing factors in selected public health facilities in South West Shoa Zone, Oromia Regional State, Ethiopia. June 2017. Available at: http://etd.aau.edu.et/bitstream/handle/123456789/1467/Esayas%20Tadesse.pdf?sequence=1&isAllowed=y.

[36] Jovanović V, Manojlović J, Jovanović D, Matic B, Đonović N. Management of pharmaceutical waste in hospitals in Serbia–challenges and the potential for improvement. Indian Journal of Pharmaceutical Education and Research. 2016;50(4):695–702.

[37] Federal Ministry of Health of Ethiopia. Health Sector Development IV (HSDP IV /2010-2015) plan. Addis Ababa, Ethiopia. October, 2010. Available at: https://phe-ethiopia.org/admin/uploads/attachment-721-HSDP%20IV%20Final%20Draft%2011Octoberr%202010.pdf.

[38] Debere MK, Gelaye KA, Alamdo AG, Trifa ZM. Assessment of the health care waste generation rates and its management system in hospitals of Addis Ababa, Ethiopia, 2011. BMC public health. 2013;13(1):28. 23311573 10.1186/1471-2458-13-28 PMC3565894

[39] Azage M, Kumie A. Healthcare waste generation and its management system: the case of health centers in West Gojjam Zone, Amhara Region, Ethiopia. Ethiopian Journal of Health Development. 2010;24(2).

[40] Meleko A, and Adane A. Assessment of Health Care Waste Generation Rate and Evaluation of its Management System in Mizan Tepi University Teaching Hospital (MTUTH), Bench Maji Zone, South West Ethiopia. Ann Rev Resear. 2018; 1(4): 555566.

[41] Rachmania IN, Basri MH. Pharmaceutical inventory management issues in hospital supply chains. doi: 10.1016/S0140-6736(08)61762-6 Management. 2013;3(1):1–5.

[42] Dwivedi S, Kothiyal P. Inventory management: A tool of identifying items that need greater attention for control. The Pharma Innovation. 2012;1(7, Part A):125.

[43] Almeida MARd Wilson AMMM, Peterlini MAS. Evaluating pharmaceutical waste disposal in pediatric units. Revista da Escola de Enfermagem da USP. 2016;50(6):922–8. 10.1590/S0080-623420160000700007 28198956

[44] Desalegn AA. Assessment of drug use pattern using WHO prescribing indicators at Hawassa University teaching and referral hospital, south Ethiopia: a cross-sectional study. BMC health services research. 2013;13(1):170. doi: 10.1186/1472-6963-13-170 23647871 PMC3651314

[45] Kagashe GA, Massawe T. Medicine stock out and inventory management problems in public hospitals in Tanzania: A case of Dar Es Salaam region hospitals. International Journal of Pharmacy. 2012;2(2):252–9.

[46] Abah SO, Ohimain EI. Healthcare waste management in Nigeria: A case study. Journal of Public health and Epidemiology. 2011;3(3):99–110.

[47] Ahmed N, Gasmelseed G, Musa A. Assessment of medical solid waste management in Khartoum state hospitals. Journal of Applied and Industrial Sciences. 2014;2(4):201–5.

[48] Uddin MN, Islam MR, Yesmin K. Knowledge on hospital waste management among senior staff nurses working in a selected medical college hospital of Bangladesh. Journal of Waste Management. 2014;2014. doi: 10.1016/j.wasman.2014.06.029 25080055

[49] Manyele S, Lyasenga T. Factors affecting medical waste management in lowlevel health facilities in Tanzania SV. African Journal of Environmental Science and Technology. 2010;4(5).

[50] Sasu S, Kümmerer K, Kranert M. Assessment of pharmaceutical waste management at selected hospitals and homes in Ghana. Waste Management & Research. 2012;30(6):625–30. doi: 10.1177/0734242X11423286 22081380

[51] Doh Manila. Health care waste managment manual. Available at: https://www.doh.gov.ph/sites/default/…/Health_Care_Waste_Management_Manual.pdf.

[52] Akum FA. An assessment of medical waste management in Bawku Presbyterian hospital of the upper east region of Ghana. Merit Research Journal of Environmental Science and Toxicology. 2014;2(2):27–38.

[53] Lessing C, Mace C, Bissell K. The availability, pricing and affordability of three essential asthma medicines in 52 low-and middle-income countries. Pharmacoeconomics. 2013;31(11):1063–82. doi: 10.1007/s40273-013-0095-9 24127259

[54] Moges F, Endris M, Belyhun Y, Worku W. Isolation and characterization of multiple drug resistance bacterial pathogens from waste water in hospital and non-hospital environments, Northwest Ethiopia. BMC research notes. 2014;7(1):215. doi: 10.1186/1756-0500-7-215 24708553 PMC4234977

[55] Shaaban H, Alghamdi H, Alhamed N, Alziadi A, Mostafa A. Environmental Contamination by Pharmaceutical Waste: Assessing Patterns of Disposing Unwanted Medications and Investigating the Factors Influencing Personal Disposal Choices. 2018.

